# Get Your Pedigree Ready

**Published:** 1897-06

**Authors:** 


### Get Your Pedigree Ready.

Who will deny that "the world do move" when we read that a score of unmarried women in New York have formed a society for the prevention of hereditary diseases? To accomplish this difficult task they have taken a solemn oath not to marry any man whose family is tainted with such a disease as consnmption, insanity or alcoholism. No one can help feeling that good results would follow the strict adherence to such a plan, but why limit it to women? Should not a young man be just as particular in choosing a wife? He should be, for disease is transmitted by one sex as well as the other.—*Pacific Medical Journal.*

